# Identification of proteins found to be significantly altered when comparing the serum proteome from Multiple Myeloma patients with varying degrees of bone disease

**DOI:** 10.1186/1471-2164-15-904

**Published:** 2014-10-17

**Authors:** Paul Dowling, Catriona Hayes, Kay Reen Ting, Abdul Hameed, Justine Meiller, Constantine Mitsiades, Kenneth C Anderson, Martin Clynes, Colin Clarke, Paul Richardson, Peter O’Gorman

**Affiliations:** Department of Biology, National University of Ireland, Maynooth, Co, Kildare, Ireland; National Institute for Cellular Biotechnology, Dublin City University, Glasnevin Dublin 9, Ireland; Mater Misericordiae University Hospital, Dublin 7, Ireland; Department of Medical Oncology, Dana-Farber Cancer Institute, Boston, USA; Department of Medicine, Harvard Medical School, Boston, USA; School of Medicine & Medical Science, University College Dublin, Dublin 4, Ireland

**Keywords:** Biomarkers, Bone disease, C4, Mass spectrometry, PON1, Proteomics

## Abstract

**Background:**

Bone destruction is a feature of multiple myeloma, characterised by osteolytic bone destruction due to increased osteoclast activity and suppressed or absent osteoblast activity. Almost all multiple myeloma patients develop osteolytic bone lesions associated with severe and debilitating bone pain, pathologic fractures, hypercalcemia, and spinal cord compression, as well as increased mortality. Biomarkers of bone remodelling are used to identify disease characteristics that can help select the optimal management of patients. However, more accurate biomarkers are needed to effectively mirror the dynamics of bone disease activity.

**Results:**

A label-free mass spectrometry-based strategy was employed for discovery phase analysis of fractionated patient serum samples associated with no or high bone disease. A number of proteins were identified which were statistically significantly correlated with bone disease, including enzymes, extracellular matrix glycoproteins, and components of the complement system.

**Conclusions:**

Enzyme-linked immunosorbent assay of complement C4 and serum paraoxonase/arylesterase 1 indicated that these proteins were associated with high bone disease in a larger independent cohort of patient samples. These biomolecules may therefore be clinically useful in assessing the extent of bone disease.

**Electronic supplementary material:**

The online version of this article (doi:10.1186/1471-2164-15-904) contains supplementary material, which is available to authorized users.

## Background

Osteolytic bone disease is a common complication of multiple myeloma, with 70% of patients having bone lesions at diagnosis [1]. Bone-destroying osteoclasts are more active in myeloma than bone-forming osteoblasts, ultimately leading to bone destruction. These lesions weaken bone, causing pain, spinal cord compression, hypercalcemia, and increased risk of fractures [2]. Moreover, bone repair appears to be inhibited at sites of osteolytic lesions. The standard method for detecting bone lesions is through a radiographic (x-ray) skeletal survey, which is relatively insensitive. Studies have demonstrated that whole-body computerised tomography (CT) scans are better than x-rays for detecting bone lesions; however, concerns about radiation dose exist [3, 4]. While radiological evaluation of the bony skeleton is important for detecting bone disease in MM patients, imaging studies only provide cross-sectional information reflecting bone dynamics. Even the most sensitive imaging techniques only detect established cortical bone destruction.

Biomolecules, measured in biofluids such as blood or urine provide for longitudinal information concerning bone disease. Indeed MM is characterised by repeated relapses, with increasing destruction at relapse [5]; sensitive markers of osteoclast destruction therefore may detect sub-clinical bone destruction and allow for early preventative intervention. Specifically, ELISA-based bone marker assays would offer a low cost, widely accessible, non-radiation-based method of longitudinally monitoring dynamic changes in osteoclast activity [6]. Current bone biomolecules can be divided into two categories, collagen fragments released from the bone matrix during resorption (degradation); and enzymes released from either osteoblasts or osteoclasts. Biomarkers reflecting osteoclast-mediated degradation of collagen include N-terminal cross-linking telopeptide of type-1 collagen (NTX), C-terminal cross-linking telopeptide of type-1 collagen (CTX), C-terminal cross-linking telopeptide of type-1 collagen generated by metalloproteinase (ICTP), and deoxypyridinoline (DPD). Procollagen type-1 N-propeptide (PINP) and procollagen type-1 C-propeptide (PICP) signify new bone formation [7, 8]. Tartrate-resistant acid phosphatase isotype 5b (TRACP-5b) is an enzyme used as a marker of osteoclast number and activity, while bone-specific alkaline phosphatase (BALP) and osteocalcin (OC), produced by osteoblasts, are used as markers of osteoblast number and activity [9]. Receptor activator of nuclear factor-kappa B ligand (RANKL) and Osteoprotegerin (OPG) are also important markers of bone turnover. RANKL increases osteoclast activity and is regulated by OPG, the soluble decoy receptor for RANKL, which serves as an inhibitor of RANKL activity. The RANKL/OPG ratio therefore can serve as an index of osteoclastogenic activity [10, 11]. Although these biochemical markers of bone destruction and formation are useful measurements in assessing the extent of myeloma bone disease, no individual marker has proved to be completely effective at mirroring the dynamics of bone disease activity [6].

In this investigation, we applied a discovery proteomics-based approach to identify novel circulating biomolecules that accurately correlate with degree of bone disease in MM patients.

## Results

### Patient data

Table 1 shows clinical data for the 111 patient samples (D - samples used in the discovery phase; V - samples used in the validation phase) used in this study including 41 patients with no bone disease, 49 patients with high bone disease, and 21 patients with MGUS/SMM (Monoclonal gammopathy of undetermined significance (MGUS) & smoldering multiple myeloma (SMM)). A total of 10 patient serum samples with no bone disease and 10 patient serum samples with high bone disease were used during the discovery phase analysis. The remaining samples, including MGUS and SMM were included in the validation analysis of C4, B100 and PON1.

**Table 1:** C**linical data**

Patient ID	No bone disease	Bisphosphonate tx (Y/N)
1 (D)	PET: no focal myelomatous lesions	Y
2 (D)	SS: No lytic lesions.	N
3 (D)	SS: no lytic lesions. Diffuse osteopenia.	N
4 (D)	SS: no evidence of lytic bone lesions	N
5 (D)	SS: no lytic disease	N
6 (D)	SS: no focal lytic lesions.	Y
7 (D)	SS and MRI spine: no lytic lesions	N
8 (D)	SS: no lytic lesions	Y
9 (D)	Notes: no evidence of bone disease on SS.	N
10 (D)	SS: no bone disease	N
11 (V)	SS: no lytic lesions identified.	N.
12 (V)	SS: no lytic lesions. MRI spine: no focal ltic lesions.	N
13 (V)	SS: no lytic lesions.	N
14 (V)	SS: no bone disease	N
15 (V)	Notes: SS negative for lytic bone disease.	N
16 (V)	SS and MRI spine: no lytic disease	N
17 (V)	SS: no lytic lesions.	N
18 (V)	SS: no lytic lesions	N
19 (V)	SS (*x*2): negative metastatic bone series.	n/a
20 (V)	SS: no lytic lesions or vert #s	N
21 (V)	SS: no evidence of lytic disease	N
22 (V)	SS: no lytic lesions	N
23 (V)	SS: no lytic lesions	Y
24 (V)	SS: no lytic lesions	N
25 (V)	SS: no lytic lesions	Y
26 (V)	SS: no lytic lesions.	N
27 (V)	SS: no dominant lytic lesion seen.	N
28 (V)	SS: no findings related to myeloma.	N
29 (V)	Notes: no lytic lesions SS	N
30 (V)	Notes (*x*2): no myeloma bone disease on SS.	N
31 (V)	Notes: no evidence of lytic bone disease (SS).	Y
32 (V)	SS: no evidence of lytic bone disease.	Y
33 (V)	SS and CT abdo/pelvis: no lytic lesions	Y
34 (V)	SS (*x*2): no lytic bone diesase.	Y
35 (V)	SS: no focal lucencies suggestive of myeloma.	n/a
36 (V)	SS: no lytic lesions	N
37 (V)	SS: no lytic lesions	Y
38 (V)	SS, CT spine and PET-CT: no lytic lesions.	N
39 (V)	SS: no lytic lesions	N
40 (V)	SS: no lytic disease	Y
41 (V)	SS: no lytic lesions	Y
**Patient ID**	**High bone disease**	**Bisphosphonate tx (Y/N)**
42 (D)	Lytic lesions in hip on MRI, and in thorax on CT.	N
43 (D)	Multiple lytic lesions, skull, pelvis, femur (SS)	Y
44 (D)	MRI: widespread bone disease, including extramedullary involvement.	Y
45 (D)	SS: multiple lucencies skull, pelcis, femora, compression #s spine.	n/a
46 (D)	SS: diffuse lytic disease	N
47 (D)	SS: C2 lesion and femoral neck lesions.	N
48 (D)	SS: multiple involving skull, humeri, femora.	Y
49 (D)	SS: multiple lucencies in the skull, and femoral lesion.	N
50 (D)	Notes: Multiple lytic bone lesions	Y
51 (D)	SS: multiple lytic lesions throughout skeleton.	Y
52 (V)	SS: lucencies invol. Ribs, scapulae, T/L spines.	N
53 (V)	Notes: multiple bony lesions with soft tissue extension.	n/a
54 (V)	MRI: multiple spinal lesions	Y
55 (V)	Notes: extensive bone involvement.	Y
56 (V)	SS: lucencies of skull, humerus, femur, pelvis.	N
57 (V)	SS: lesions of skull, ribs, humera, femur.	N
58 (V)	SS: multiple lucencies femurs, humeri, skull	Y
59 (V)	SS and MRI: skull, clavicle, vertebral lesions.	Y
60 (V)	Vertebral, femoral, extramedullary (lung) lesions.	N
61 (V)	SS: skull, humeri, vert lesions.	Y
62 (V)	PET-CT: increased uptake left ilial and T12 vert, rib #s	N
63 (V)	MRI: multiple compression #s spine, sacral lesions	Y
64 (V)	SS: multiple lucencies, skull, humeri, femurs.	n/a
65 (V)	SS: femurs, pelvis, skull luc, compressn # T9	Y
66 (V)	CT: lumbar spine, rib, pelvis, sternal lesions	Y
67 (V)	MRI: multiple vertebral lesions	Y
68 (V)	SS: lesions skull, pelvis, femora.	Y
69 (V)	SS: skull, humrea, vert lesions	n/a
70 (V)	Notes: scapular, pelvic and hip lesions.	Y
71 (V)	SS: skull, vert, humeri lucencies	N
72 (V)	SS/MRI: multiple lesions pelvis and lt humerus.	Y
73 (V)	SS/MRIs: lumbar, thoracic and sacral lesions.	Y
74 (V)	SS/PET: skull lesions and sternal plasmacytoma	N
75 (V)	SS: lucencies femora, skull, humerus.	N
76 (V)	SS: diffuse myel. involve. (skull, hum, fem, t spine, skull).	Y
77 (V)	Notes: lucencies C/T spine and scapula.	N
78 (V)	Notes: destructive lesions L2 and acetabulum.	Y
79 (V)	SS: compressn #s spine, lucencies skull, femora, humeri.	Y
80 (V)	PETCT: diffuse uptake spine, sternum, femora.	N
81 (V)	MRI: T7-9 lesions with cord compression	N
82 (V)	SS: lucencies femora, skull, humeri.	Y
83 (V)	SS: lucencies of skull, clavicles, humera, T spine.	Y
84 (V)	CT: 2 rib #s and compressn # T5	N
85 (V)	CT: innumerable lesions within axial and appendicular skeleton.	N
86 (V)	SS: lucencies in vert, pelvis, humeri, femora.	N
87 (V)	SS: lesions in skull, ribs, T spine, humeri.	Y
88 (V)	SS: multiple lesions skull, pelvis, and T12 compressn #.	n/a
89 (V)	Notes: review of outside films demonstrates extensive bony disease	N
90 (V)	SS: Numerous lesions in skull and extremeties.	N
**Patient ID**	**Pre-malignant myeloma**	**Bisphosphonate tx (Y/N)**
91 (V)	MGUS	N
92 (V)	MGUS	N
93 (V)	MGUS	N
94 (V)	MGUS	N
95 (V)	MGUS	N
96 (V)	MGUS	N
97 (V)	MGUS	N
98 (V)	MGUS	N
99 (V)	MGUS	N
100 (V)	SMM	N
101 (V)	SMM	N
102 (V)	SMM	N
103 (V)	SMM	N
104 (V)	SMM	N
105 (V)	SMM	N
106 (V)	SMM	N
107 (V)	SMM	N
108 (V)	SMM	N
109 (V)	SMM	N
110 (V)	SMM	N
111 (V)	SMM	N

Clinical data for the 111 patient samples used in this study. 41 patients diagnosed with no bone disease, 49 patients with high bone disease and 21 patients with monoclonal gammopathy of undetermined significance (MGUS) or smoldering multiple myeloma (SMM). Samples are labeled with (D) for discovery and (V) for validation, indicating the phase in which they were used. Information on whether the patients were on bisphosphonate treatment (tx) at the time the sample was taken is included (Y = yes, N = no). n/a = not available.

### Label-free mass spectrometry

In total 159 proteins were positively identified in the Proteominer enriched serum samples using Progenesis LC-MS to align these runs, of which 24 differentially expressed proteins are included in the protein table (Table 2). Label-free mass spectrometry is a powerful and widely-used technique for identifying and quantifying relative changes in complex protein samples, including serum. Serum presents a significant challenge in proteomics research, displaying a huge dynamic range which exceeds ten orders of magnitude. To help overcome this problem, ProteoMiner™ pre-fractionation was employed to compress the dynamic range and facilitate the discovery of disease-related biomolecules found in the serum proteome. Of the proteins identified during the discovery phase, Complement C4-A (C4A, p =0.05, 2.8-fold ↑ high bone disease), Apolipoprotein B-100 (APOB, p =0.04, 2.2-fold ↓ high bone disease) and Serum paraoxonase/arylesterase (PON1, p =0.02, 1.6-fold ↑ high bone disease) were selected for further analysis in a larger cohort of patient samples. Previous work by our group has demonstrated that serum levels of C4, B100 and PON1 are abnormal in patients found to be responders/non-responders to MM therapies, including Thalidomide and Bortezomib (see Additional file 1: Table S1), therefore it would be of interest to see if any correlation existed with bone disease, especially as these proteins appeared in the discovery phase protein list.

**Table 2:** **Protein list**

Gene symbol	Protein identification	Measured peptides	MASCOT score	BH adjusted p-value	Fold change
VTN	Vitronectin	6	391	0.02	-2.7
KNG1	Kininogen-1	2	115	0.02	1.4
THBS1	Thrombospondin-1	5	343	0.02	3.0
PON1	Serum paraoxonase/arylesterase 1	3	166	0.02	-1.6
PLG	Plasminogen	8	545	0.02	1.5
F5	Coagulation factor V	4	210	0.03	1.8
F2	Prothrombin	10	722	0.03	1.8
AMBP	Protein AMBP	2	124	0.04	2.4
APOB	Apolipoprotein B-100	4	1640	0.04	2.2
PROS1	Vitamin K-dependent protein S	3	198	0.04	2.0
CFHR1	Complement factor H-related protein 1	2	171	0.04	1.8
TTR	Transthyretin	2	163	0.04	-2.2
PF4	Platelet factor 4	2	186	0.04	1.4
SERPINA1	Alpha-1-antitrypsin	3	247	0.04	2.4
AHSG	Alpha-2-HS-glycoprotein	5	454	0.04	3.5
FN1	Fibronectin	7	567	0.05	-4.5
HRG	Histidine-rich glycoprotein	2	97	0.05	3.0
TF	Serotransferrin	2	195	0.05	3.2
IGHG2	Ig gamma-2 chain C region	3	214	0.05	2.0
ALB	Serum albumin	4	311	0.05	3.6
C1S	Complement C1s subcomponent	2	112	0.05	2.6
IGHM	Ig mu chain C region	4	265	0.05	-2.7
C4A	Complement C4-A	5	310	0.05	-2.8
A2M	Alpha-2-macroglobulin	2	157	0.05	3.3

List of differentially expressed proteins comparing no bone disease to high bone disease. The table includes information on gene symbol, protein identification, measured peptides (used for quantitation), confidence score (MASCOT), Benjamini-Hochberg adjusted p-value and fold-change (- indicates a protein is decreased in abundance in no bone disease compared to high bone disease, all other proteins are increased in abundance in no bone disease compared to high bone disease). A total of 10 patient serum samples with no bone disease and 10 patient serum samples with high bone disease were used during the discovery phase analysis.

### STRING analysis

**Figure 1:**
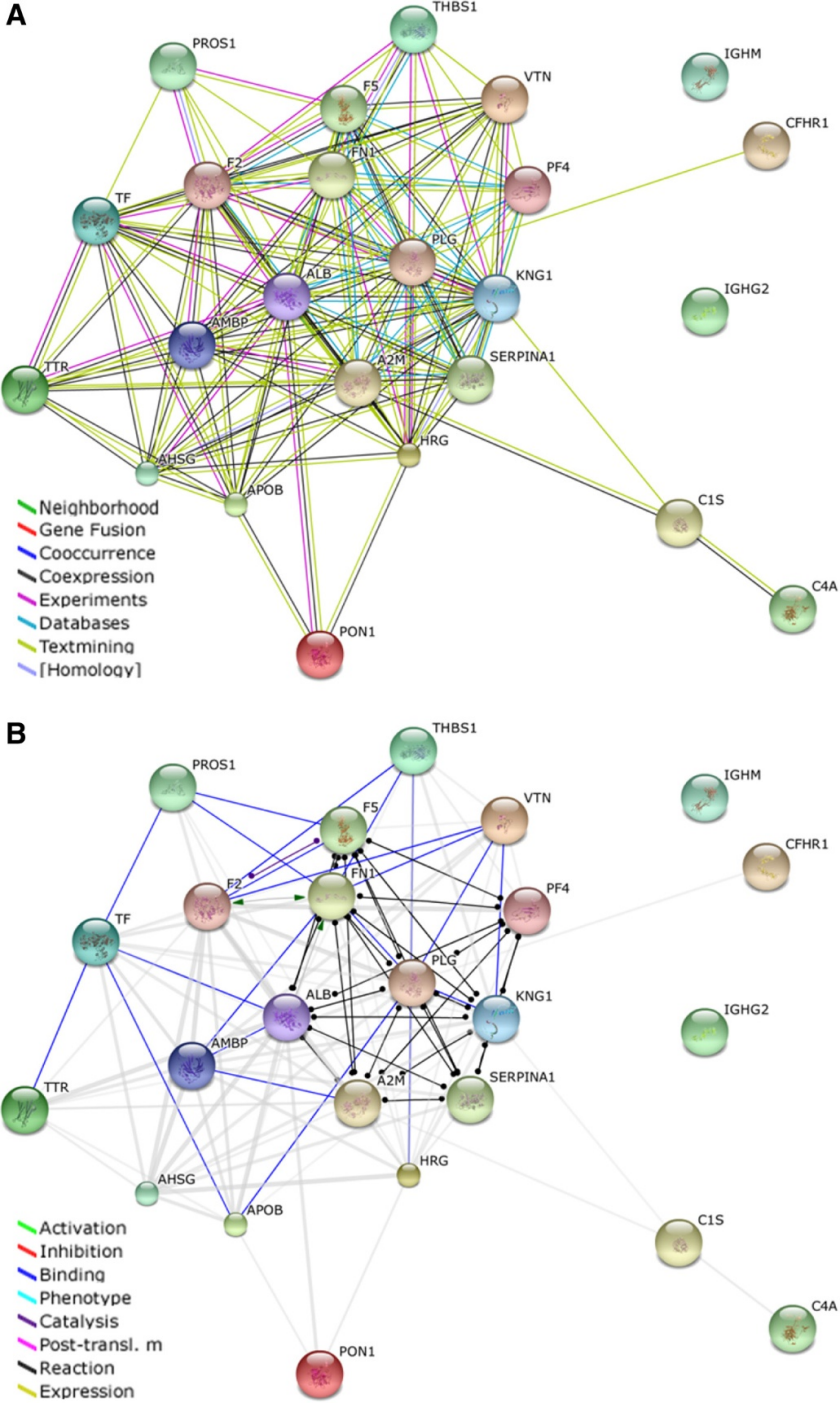
**STRING analysis of 24 differentially expressed proteins listed in Table** 2 **.** The STRING program generates functional protein association networks. **(A)** Evidence view; uses different-coloured lines to depict the type of evidence that supports each interaction. **(B)** Action view; uses different-coloured lines to depict the types of interaction between proteins.

Protein associations and networks from the list of proteins with significantly changed abundance levels between no bone disease and high bone disease MM patient serum samples were analysed using STRING (Search Tool for the Retrieval of Interacting Genes), a database of known and predicted protein-protein interactions [12]. It generates a network of interactions from a variety of sources, including different interaction databases, text mining, genetic interactions, and shared pathway interactions. The evidence view is shown in Figure 1A, and the action view is shown in Figure 1B. The colour-coded legend for the evidence view shows the type of the interactions between proteins including databases and text mining. The action view shows the type of evidence that supports the interaction, such as binding and inhibition. A central cluster of interacting proteins is evident comprised of Alpha-1-antitrypsin (SERPINA1 ↓), Albumin (ALB ↓), Plasminogen (PLG ↓), Kininogen 1 (KNG1 ↓), Coagulation factor V (F5 ↓), Fibronectin (FN1 ↑), Alpha-2-macroglobulin (A2M ↓) and Platelet factor 4 (PF4 ↓). The majority of the proteins in this cluster were found to be decreased in abundance in high bone disease MM patient samples compared to no bone disease, except for FN1.

### Box and whisker plots and area under the curve (AUC) analysis

**Figure 2:**
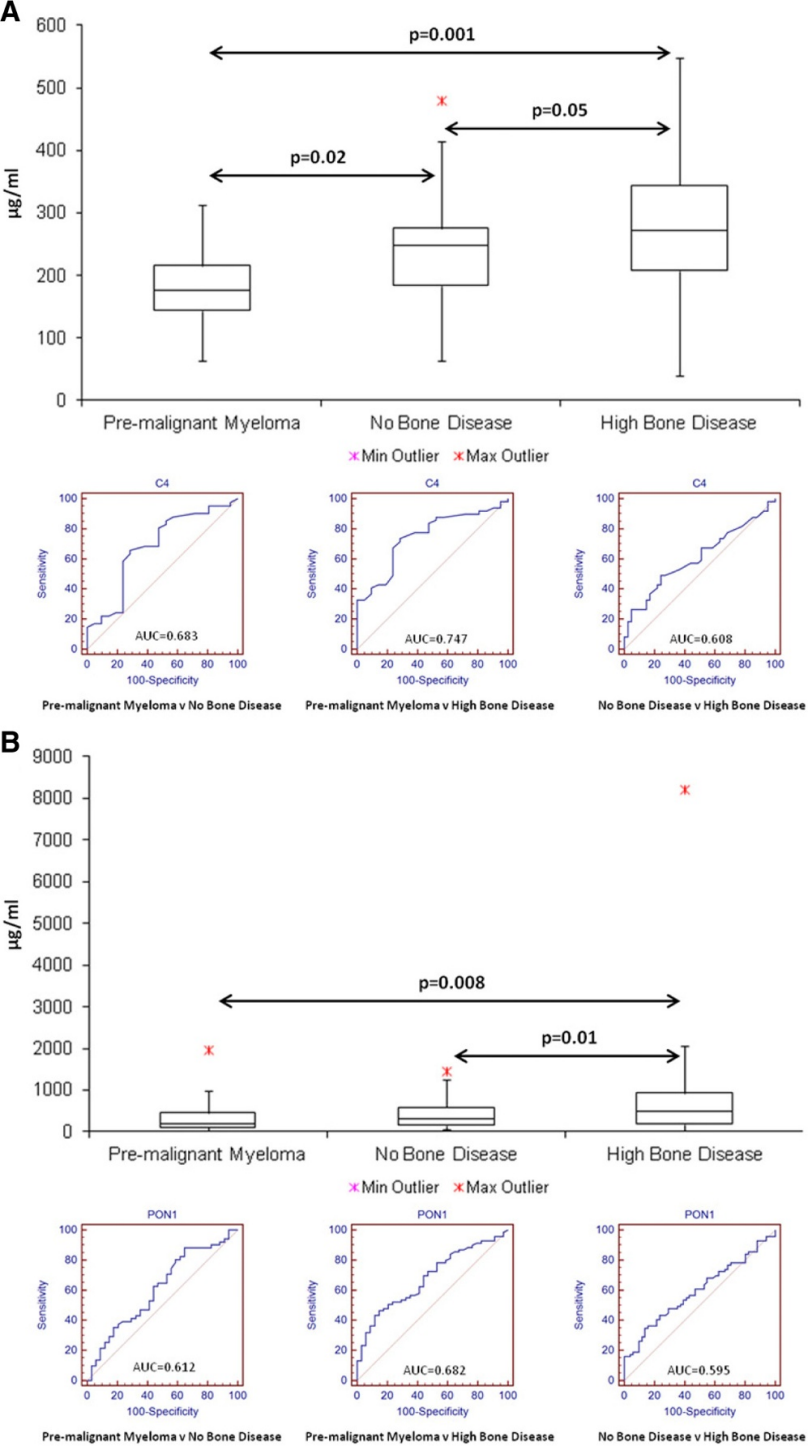
**Box and whisker plots and ROC curves with associated AUC-values for Complement C4 (A) and PON1 - Serum paraoxonase/arylesterase (B) in patient samples (Pre-malignant Myeloma: MGUS/SMM, MM: No Bone Disease and MM: High Bone Disease).** The box and whisker plots display information on the range, median and quartiles. A total of 31 patient samples with no bone disease, 39 patient samples with high bone disease and 21 patient samples with MGUS/SMM were screened by ELISA.

Box and whisker plots were constructed to display information about the range, the median and the quartiles for Complement C4 (Figure 2A) and Serum paraoxonase/arylesterase (Figure 2B) based on ELISA results from an independent validation cohort of patient samples, including monoclonal gammopathy of undetermined significance (MGUS), smoldering multiple myeloma (SMM), MM patients with no bone disease and MM patients with bone disease. B100 was not statistically significantly correlated with bone disease in the independent sample set. C4 and PON1 were found to be significant, with overall concentration increasing from MGUS/SMM (C4: 176 ug/ml, PON1: 204 ug/ml) to no bone disease (C4: 248 ug/ml, PON1: 301 ug/ml) to high bone disease (C4: 272 ug/ml, PON1: 502 ug/ml). C4 was found to be statistically correlated in 3 comparisons: p =0.02 (MGUS/SMM v no bone disease), p =0.001 (MGUS/SMM v high bone disease) and p =0.05 (no bone disease v high bone disease). PON1 was significantly associated in 2 comparisons: p =0.008 (MGUS/SMM v high bone disease) and p =0.01 (no bone disease v high bone disease). Figure 2 summarises information on the median, minimum and maximum values for C4 and PON1. Statistical analysis was also completed comparing patients who were receiving bisphosphonate treatment (tx) versus no treatment at the time the sample was obtained (Table 1) for C4 and PON1. None of these comparisons were found to be statistically significant (data not shown).

Receiver operating characteristic (ROC) curve were constructed with the area under the ROC curve (AUC) calculated to indicate the performance of both C4 and PON1 at distinguishing pre-malignant myeloma v no bone disease, pre-malignant myeloma v high bone disease and no bone disease v high bone disease (Figure 2). The most significant AUC-values were found when comparing pre-malignant myeloma to high bone disease for both C4 and PON1, with values of 0.747 and 0.682 respectively. AUC-values were also determined for the combination of C4 and PON1 using logistic regression analysis to discriminate between the different groups, with a value of 0.695 for pre-malignant myeloma v no bone disease, 0.801 for pre-malignant myeloma v high bone disease and 0.702 for no bone disease v high bone disease. Similarly to the individual AUC-values, the most significant value was found for the pre-malignant myeloma v high bone disease comparison (0.801).

## Discussion

Protein analysis, specifically looking at serum proteins found to have differences in abundance levels associated with disease phenotype, in this case pre-malignant MM and MM patients diagnosed with no bone disease or high bone disease, can help delineate the complex mechanisms of bone biology and also provide clinicians with a suite of biomarkers to aid in the management of MM patients. In this investigation, ProteoMiner fractionated patient samples were analysed using a bottom-up (peptide level) label-free mass spectrometric proteomic strategy for biomarker discovery. A number of proteins found to be statistically significant, correlating with bone disease, were identified including enzymes, extracellular matrix glycoproteins, and members of the complement system. C4, B100 and PON1 were selected for further analysis in a larger independent cohort of patient samples (based on previous unpublished findings), with both C4 and PON1 confirmed to be significantly associated with bone disease.

MM is characterised by repeated relapses ultimately culminating in fatal refractory disease. The rate of MM bone disease increases with progression of disease. While CT and MRI scans have improved resolution and sensitivity for detecting bone disease, there is a need for more sensitive biochemical markers that will detect sub-clinical bone disease, [13]. Protein markers of bone turnover provide clinically useful supportive information on bone homeostasis by measuring enzymes, cytokines and collagen breakdown products released during bone formation and of degradation products produced during bone resorption. A range of biochemical bone markers are available that allow a specific and sensitive evaluation of the rate of bone formation and bone resorption of the skeleton; however, none of the currently known biomarkers are used in clinical practice, and more robust and sensitive biomarkers are needed to both monitor bone turnover and aid in managing patients with bone disease [14]. Due to expense, access to radiology, and radiation exposure, radiology techniques are used in the setting of rising paraprotein or new symptomatology. The majority of patients will already have new bone lesions and/or fracture [15]. Moreover, there is a concern that existing bone markers are not interpretable in patients receiving bisphosphonate therapy [16]. These challenges reinforce the need for non-invasive, informative, and sensitive bone markers that will allow for early detection of bone disease and preventative intervention.

It was observed that complement factor H-related protein 1 and complement C1s subcomponent were present at lower concentrations in the patients with high bone disease compared to no bone disease. The complement system is made up of a series of about 25 proteins, two-thirds of which circulate in the plasma while the remainder are present on cell and tissue surfaces. A recent review discusses the link between the complement proteins and their role in bone development and homeostasis, specifically investigating their influence on osteoblast and osteoclast activity [17]. The complex interplay between these systems is still not fully understood but increasing evidence points to the complement anaphylatoxins influence the migration of bone cells and the controlled interactions between osteoblast and osteoclast. C4, as a major protein of the classical cascade potentially has an important role in these important interactions. This system works to “complement” the activity of antibodies in destroying bacteria, either by facilitating phagocytosis or by puncturing the bacterial cell membrane; however, recent developments have shed some light on the role of the complement system in bone formation and maintenance. We for the first time found Complement C4-A to be increased in the high bone disease patient cohort. In the larger validation cohort, increasing levels of complement C4 were determined when moving from MGUS/SMM to no-bone disease to high-bone disease phenotypes. Increased C4 values have previously been associated with systemic lupus erythematous, rheumatoid arthritis, severe bacterial or viral infections, cancer, and myocardial infarction [18–20]. Zheng and co-workers reported increased levels of complement C4 in MM patients, suggesting that upregulation of complement components is due to defective activation [21].

Both Apolipoprotein B100 and PON1 were selected for further analysis in a larger sample set. B100 was not found to be significant in the validation cohort, but PON1 did show a stepwise increase in concentration from MGUS/SMM to high bone disease (as did complement C4). Serum paraoxonase/arylesterase 1 (PON1) degrades chemicals including several types of organophosphate pesticides and pharmaceutical drugs in the body. PON1 also has antioxidant properties, protecting both high- and low-density lipoproteins from oxidation associated with changing mineral content and decreased formation of bone [22]. Polymorphisms and abnormal enzymatic activity of PON1 have also been implicated in heart disease, osteoporosis, atherosclerosis, and cancer [23–26]. Moreover, PON1 can directly suppress the macrophage pro-inflammatory response [27]. The PON1 gene is activated by peroxisome proliferator-activated receptor γ (PPARγ), a member of the ligand-activated nuclear receptor superfamily [28]. Redox balance in bone cells is a key modulator of normal functioning, and the increased levels of PON1 detected in patient serum samples in this study may therefore represent a protective mechanism against the effects of oxidative stress on bone formation associated with osteoclastic resorption. Oxidative stress is involved in the pathogenesis of bone diseases such as osteoporosis, increase in osteoclasts activity and upregulation of osteoclast differentiation. Therefore, it could be proposed that the increased levels of PON1 are in response to this increased stress, to facilitate the antioxidant bioscavenger of this molecule and try to re-balance bone remodelling.

A number of other proteins were found to be significantly correlated with different levels of bone disease. Cells adhere to the extracellular matrix through interaction with adhesive extracellular matrix glycoproteins, including vitronectin (VTN) and Fibronectin (FN1). VTN, also known as S-protein, is a multi-functional plasma and extracellular matrix protein with defined activity in cell adhesion, thrombosis, complement activation, fibrinolysis, inflammation, and platelet adhesion. Several groups have shown the important role that vitronectin plays in the development and regulation of bone structure [29–31]. FN1 is a multifunctional, extracellular matrix glycoprotein composed of two disulfidebound polypeptides of molecular weight 220 kDa exhibiting structural and adhesive properties in cell-associated fibrillar matrices. Similar to VTN, FN1 is implicated in early stages of bone formation, and continuous presence of FN1 is required for maintaining the integrity of the bone matrix [32–34]. Our discovery phase results demonstrate a significant elevation in the concentrations of both VTN and FN1 in high bone disease compared to no bone disease patient samples, suggesting that these proteins are released into the circulation due to increased bone resorption related to stimulation of osteoclast formation and activity. These results compare with the distribution of type I collagen fragments after osteoclastic degradation, including NTX and CTX, markers which are used for the early diagnosis of bone lesions [35]. Finally, bisphosphonate treatment at the time of sample collection was an important consideration with respect to the potential impact that this treatment would have on expression of the candidate biomarkers. Statistical analysis showed that bisphosphonate treatment did not significantly impact C4 and PON1 levels, suggesting their potential utility in patients receiving bisphosphonate therapy, in combination with existing biochemical tests and MRI/CT scanning.

From the information in the supplementary table, both C4A and PON1 were found to be elevated in patients responding to treatment, while in this study, both were found to increase in concentration in the high bone disease group compared to both the pre-malignant myeloma and no bone disease cohorts. Therefore, a direct link between disease burden and the levels of these candidate biomarkers is not obvious and may indicate a more defined role in measuring bone disease. A limitation of this investigation is the number of groups analysed in the validation phase. Pre-malignant myeloma and both no/high bone disease cohorts were examined. Ideally this would be expanded out to include multiple myeloma disease stages with associated bone disease information. Also, the main utility of these proposed biomarkers would be in tracking individual patients during the course of their disease, investigate how the abundance of these biomarkers changes in relation to disease state and how this information could be useful in patient management.

## Conclusion

In summary, this study identifies novel candidate biomarkers associated with bone disease in MM patients. These novel biomarkers are currently being further evaluated in prospective studies of MM bone disease to assess their clinical utility.

## Methods

### Patients selection and sample collection

The participating subjects, attending the Jerome Lipper Multiple Myeloma Center and LeBow Institute for Myeloma Therapeutics, Dana-Farber Cancer Institute, Harvard Medical School, Boston, MA, US, and the Mater Hospital, Dublin, Ireland, gave written informed consent in accordance with the Declaration of Helsinki that was approved by the Institutional Review Board (Dana-Farber Cancer Institute) and the Mater Hospital ethics committee. The samples were collected according to standard phlebotomy procedures from consented patients. 10 ml of blood was collected into additive free (serum) blood tubes and was allowed to clot for 30 min to 1 hr at room temperature. The serum was denuded by pipette from the clot and poured into a clean tube. The tubes were centrifuged at 1000 × g for 30 min at 4°C. Serum was aliquoted in the cryovial tubes, labeled and stored at -80°C until time of analysis. The time from sample procurement to storage at -80°C was less than 3 hr. Each serum sample underwent not more than three freeze/thaw cycles prior to analysis.

### ProteoMiner™ fractionation

Serum protein equalization was performed using ProteoMiner™ enrichment kit according to the manufacturer procedure. In summary, the storage solution was first washed out from the spin column containing 100 μL of peptide beads with deionised water. Thereafter, the column was washed with the 10 mM NaH_2_PO_4_, 150 mM NaCl, pH 7.4 solution provided with the kit. When the spin column was ready for sample binding, 1 mL of centrifuged serum sample was added to the column and equilibrated at room temperature for 2 hr. The unbound proteins were removed with the wash buffer and the captured proteins were eluted by 3 × 100 μL of 8 M urea containing 2% CHAPS dissolved in 5% acetic acid.

Following vortexing, sonication and centrifugation, the protein concentration of no bone disease and high bone disease patient samples was determined. Volumes of protein suspensions were equalized using label-free solubilisation buffer and then reduced for 30 min with 10 mM DTT and alkylated for 20 min in the dark with 25 mM iodoacetamide in 50 mM ammonium bicarbonate. The proteolytic digestion of proteins was carried out in 2 steps. Firstly, digestion was performed with sequencing grade Lys-C at a ratio of 1:100 (protease/protein) for 4 hr at 37°C, followed by diluted with 4 times the initial sample volume in 50 mM ammonium bicarbonate. Secondly, further digestion was based on incubation with sequencing grade trypsin at a ratio of 1:25 (protease/protein) overnight at 37°C. The protease-treated serum protein suspensions were diluted 3:1 (v/v) with 2% trifluoroacetic acid in 20% acetonitrile. To ensure an even suspension of peptides, the samples were briefly vortexed and sonicated.

### Label-free LC-MS/MS analysis

The nano LC-MS/MS analysis of no bone disease versus high bone disease patient samples was carried out with the help of an Ultimate 3000 nanoLC system (Dionex) coupled to a an LTQ Orbitrap XL mass spectrometrer (Thermo Fisher Scientific, Dublin, Ireland) in the Proteomics Facility of the National Institute for Cellular Biotechnology, Dublin City University. The optimized methodology has been as previously described in detail. Peptide mixtures (5 μL volume) were loaded onto a C18 trap column (C18 PepMap, 300 μm id × 5 mm, 5 μm particle size, 100 Å pore size; Dionex). Desalting was achieved at a flow rate of 25 μL/min in 0.1% TFA for 10 min. The trap column was switched on-line with an analytical PepMap C18 column (75 μm id × 500 mm, 3 μm particle and 100 Å pore size; Dionex). Peptides generated from muscle proteins were eluted with the following binary gradients: solvent A (2% ACN and 0.1% formic acid in LC-MS grade water) and 0–25% solvent B (80% ACN and 0.08% formic acid in LC-MS grade water) for 240 min and 25–50% solvent B for a further 60 min. The column flow rate was set to 350 nL/min. Data was acquired with Xcalibur software, version 2.0.7 (Thermo Fisher Scientific). The MS apparatus was operated in data-dependent mode and externally calibrated. Survey MS scans were acquired in the Orbitrap in the 300–2000 *m/z* range with the resolution set to a value of 30 000 at *m/z* 400 and lock mass set to 445.120025 u. CID fragmentation was carried out in the linear ion trap with the three most intense ions per scan. Within 60 sec, a dynamic exclusion window was applied. Normalised collision energy of 35%, an isolation window of 3 *m/z* and one microscan were used to collect suitable tandem mass spectra.

### Quantitative profiling by label-free LC-MS/MS analysis

Processing of the raw data generated from LC-MS/MS analysis was carried out with Progenesis label-free LC-MS software (version 3.1; Non-Linear Dynamics, Newcastle upon Tyne, UK). Data alignment was based on the LC retention time of each sample, allowing for any drift in retention time given and adjusted retention time for all runs in the analysis. A reference run was established with the sample run that yielded most features (i.e. peptide ions). The retention times of all of the other runs were aligned to this reference run and peak intensities were then normalised. Prior to exporting the MS/MS output files to MASCOT (http://www.matrixscience.com) for protein identification, a number of criteria were employed to filter the data including: (i) peptide features with ANOVA <0.05 between experimental groups, (ii) mass peaks (features) with charge states from +2, +3, and (iii) greater than one isotope per peptide. A MASCOT generic file was generated from all exported MS/MS spectra from Progenesis software. The MASCOT generic file was used for peptide identification with MASCOT (version 2.2) and searched against the UniProtKB-SwissProt database (downloaded in January 2013) with 16,638 proteins (taxonomy: *Homo sapiens).* The following search parameters were used for protein identification: (i) MS/MS mass tolerance set at 0.5 Da, (ii) peptide mass tolerance set to 20 ppm, (iii) carbamidomethylation set as a fixed modification, (iv) up to two missed cleavages were allowed and (v) methionine oxidation set as a variable modification. For further consideration and re-importation back into Progenesis LC-MS software for further analysis, only peptides with ion scores of 40 and above were chosen. Importantly, the following criteria were applied to assign a serum associated proteins as properly identified: (i) an ANOVA score between experimental groups of ≤0.05, (ii) proteins with ≥2 peptides matched and (iii) a MASCOT score >40.

### Enzyme-linked immunosorbent assay

Serum samples were screened using different enzyme-linked immunosorbent assays for Human Complement C4 ELISA Kit (EC2102-1, Assaypro), Human Apolipoprotein B (EA7001-1, Assaypro) and Human Paraoxonase 1 (PON1) (SK00141-01, Aviscera Bioscience, Inc). Both Apo B100 and PON1 were assayed using a quantitative sandwich enzyme immunoassay technique, while C4 was measured using a quantitative competitive enzyme immunoassay technique.

### Statistical analysis

Proteins were considered to be differentially expressed upon an observation of a fold change of ≥1.4 in both directions and a Benjamini-Hochberg adjusted p-value used with all proteins ≤0.05 selected. ELISA data was analysed by constructing a standard curve as a result of plotting the mean absorbance for each standard on the x-axis against the concentration on the y-axis and draw a best fit curve through the points on the graph. Any outliers present in ELISA readings (*) were determined using Tukey’s method which considers values at a distance of 1.5 times the interquartile range (IQR) below Q1 (quartile one) or at 1.5 times the IQR above Q3 (quartile three). The ROC plots were obtained by plotting all sensitivity values (true positive fraction) on the y-axis against their equivalent (1-specificity) values (false positive fraction) for all available thresholds on the x-axis (MedCalc, version 13-0-0-0 64-bit, Medcalc Software, Mariakerke, Belgium). The area under the curve (AUC) was calculated to provide a summary of overall classifier effectiveness. For multivariate analysis of biomarker combinations, logistic regression (LR) analysis of the serum biomarker levels in these patients groups was performed.

## Authors’ information

Paul Richardson and Peter O’Gorman: Joint senior authors.

## Electronic supplementary material

Additional file 1: Table S1: Proteomics Data for Responders v Non-Responders to Bortezomib and Thalidomide. (DOCX 32 KB)

## Abbreviations

ELISA: Enzyme-linked immunosorbant assay)
HPLC: High-performance liquid chromatography
MS/MS: Tandem mass spectrometry
*m/z*: Mass-to-charge ratio.
