# Supplementary material for: Identification of proteins found to be significantly altered when comparing the serum proteome from Multiple Myeloma patients with varying degrees of bone disease
Source: BMC Genomics. 2014 Oct 17;15(1):904. doi: 10.1186/1471-2164-15-904 (PMC4213504; doi:10.1186/1471-2164-15-904)
Supplement: Supplementary file 1 — Additional file 1: Table S1: Proteomics Data for Responders v Non-Responders to Bortezomib and Thalidomide. (DOCX 32 KB) [file 12864_2014_6607_MOESM1_ESM.docx]

Table S1: Proteomics Data for Responders v Non-Responders to Bortezomib and Thalidomide

Proteomics Data (CO4A, APOB and PON1) for Responders v Non-Responders to Bortezomib and Thalidomide. Responders/Non-Responders determination based on day 100 restaging results according to the IMWG uniform response criteria for MM. The table includes information on gene name, measured peptides (used for quantitation), confidence score (MASCOT), Anova (p-value), fold-change, highest/lowest mean and protein description. IMWG: International Myeloma Working Group
